# Studies on the morphology and spreading of human endothelial cells define key inter- and intramolecular interactions for talin1

**DOI:** 10.1016/j.ejcb.2010.05.003

**Published:** 2010-09

**Authors:** Petra M. Kopp, Neil Bate, Tania M. Hansen, Nicholas P.J. Brindle, Uta Praekelt, Emmanuel Debrand, Stacey Coleman, Daniela Mazzeo, Benjamin T. Goult, Alexandre R. Gingras, Catrin A. Pritchard, David R. Critchley, Susan J. Monkley

**Affiliations:** a Department of Biochemistry, University of Leicester, Lancaster Road, Leicester LE1 9HN, UK; b Department of Cardiovascular Sciences, Robert Kilpatrick Clinical Sciences Building, Leicester, LE2 7LX, UK

**Keywords:** FA, focal adhesions, FB, fibrillar adhesions, FERM, band 4.1, ezrin, radixin, moesin, C-ABS, C-terminal actin-binding site, HUVEC, human umbilical vein endothelial cells, Talin, Integrins, Focal adhesions, Actin cytoskeleton

## Abstract

Talin binds to and activates integrins and is thought to couple them to cytoskeletal actin. However, functional studies on talin have been restricted by the fact that most cells express two talin isoforms. Here we show that human umbilical vein endothelial cells (HUVEC) express only talin1, and that talin1 knockdown inhibited focal adhesion (FA) assembly preventing the cells from maintaining a spread morphology, a phenotype that was rescued by GFP-mouse talin1. Thus HUVEC offer an ideal model system in which to conduct talin structure/function studies. Talin contains an N-terminal FERM domain (comprised of F1, F2 and F3 domains) and a C-terminal flexible rod. The F3 FERM domain binds β-integrin tails, and mutations in F3 that inhibited integrin binding (W359A) or activation (L325R) severely compromised the ability of GFP-talin1 to rescue the talin1 knockdown phenotype despite the presence of a second integrin-binding site in the talin rod. The talin rod contains several actin-binding sites (ABS), and mutations in the C-terminal ABS that reduced actin-binding impaired talin1 function, whereas those that increased binding resulted in more stable FAs. The results show that both the N-terminal integrin and C-terminal actin-binding functions of talin are essential to cell spreading and FA assembly. Finally, mutations that relieve talin auto-inhibition resulted in the rapid and excessive production of FA, highlighting the importance of talin regulation within the cell.

## Introduction

Integrin-mediated cell adhesion, spreading and migration involve a complex interplay between the signalling pathways that regulate the affinity and avidity of integrins for extracellular matrix proteins, integrin recycling and the structure and dynamics of the actin and microtubule networks (Vicente-Manzanares et al., 2009). The large adaptor protein talin plays a key role in this regard, and is part of the mechanism by which integrins are switched from the low to high affinity state (Calderwood, 2004), and it also provides a physical link between integrins and F-actin (Critchley, 2009). Talin was originally discovered as a component of cell-matrix junctions (focal adhesions; FA) in cells in culture (Burridge and Connell, 1983), and manipulation of talin function using a variety of approaches have confirmed that it is required for both cell spreading and FA assembly (Albiges-Rizo et al., 1995; Nuckolls et al., 1992; Priddle et al., 1998; Zhang et al., 2008).

Talin (270 kDa, 2541 amino acids) is a member of the FERM domain family of proteins, and substantial progress has been made in establishing how it interacts with integrins, F-actin and the FA protein vinculin (Critchley, 2009). The N-terminal talin head contains a FERM domain, and like other FERM domains, it is made up of F1, F2 and F3 domains, although it is atypical in that the F1 domain contains a large unstructured loop and is preceded by an additional domain F0. Interestingly, this domain organisation is very similar to that of the kindlin family of FERM proteins (Goult et al., 2009b) that synergise with talin to activate integrins (Moser et al., 2009). Structural studies show that the talin F3 PTB-like domain binds to both the membrane proximal NPxY motif and the helical region in β-integrin tails (Anthis et al., 2009; Garcia-Alvarez et al., 2003; Wegener et al., 2007), and mutations in F3 that disrupt these interactions abolish the ability of the talin FERM domain to activate integrins. However, it has also become apparent that basic regions in the F1 loop (Goult et al., 2010), F2 and F3 (Anthis et al., 2009; Saltel et al., 2009; Wegener et al., 2007) that interact with acidic phospholipids are also required for integrin activation in vitro.

The talin FERM domain is linked through an apparently unstructured region to the large ∼2000 amino acids flexible talin rod, which is made up of a series of 4- and 5-amphipathic helical bundles (Critchley, 2009). The rod contains a second integrin-binding site (IBS2) of unknown function (Gingras et al., 2009; Rodius et al., 2008), numerous vinculin-binding sites (Gingras et al., 2005) and at least two actin-binding sites (Hemmings et al., 1996), the best characterised of which is at the C-terminus (C-ABS) (Gingras et al*.*, 2008; Smith and McCann, 2007). It comprises a 5-helix bundle followed by a helix that mediates talin dimer formation (Gingras et al., 2008), and only the dimeric C-ABS binds efficiently to F-actin. Finally, talin is thought to exist in both an active open conformation and a more compact auto-inhibited form that is in part due to binding of the F3 FERM domain to a 5-helix bundle (residues 1655–1822) in the talin rod (Goksoy et al., 2008; Goult et al., 2009a). Significantly, this interaction masks the integrin-binding site in F3, and both the small GTPase Rap1 (Han et al., 2006; Lee et al., 2009) and PIP2 (Goksoy et al., 2008; Martel et al., 2001) have been implicated in activating talin.

Studies on talin in vertebrates at the cellular and organismal levels are complicated by the existence of two talin genes that encode similar proteins (Debrand et al., 2009). Although the functional differences between the two isoforms have not been established, loss of talin1 is embryonic lethal at gastrulation (Monkley et al., 2000) whereas talin2 knockout mice are viable and fertile (Chen and Lo, 2005). However, talin2 appears to compensate for loss of talin1 in cells in culture, and fibroblasts derived from *Tln1*^−/−^ ES cells upregulate talin2, and are able to spread and assemble FA (Priddle et al., 1998; Zhang et al., 2008). We therefore sought to identify a cell type that was easy to grow and transfect, and in which we could knockout or knockdown talin1 without upregulation of talin2. In the present study, we have shown that human umbilical vein endothelial cells (HUVEC) fulfil these criteria. Thus HUVEC express only talin1, and siRNA-mediated knockdown of human talin1 results in a reduction in cell spreading and FA assembly, a phenotype that can be rescued by expression of mouse GFP-tagged talin1. This has enabled us to conduct a comprehensive analysis of the function of talin1 as it relates to the structure of the protein.

## Results

### Knockout of talin1 in mouse fibroblasts rapidly leads to upregulation of talin2

**Fig. S1:**
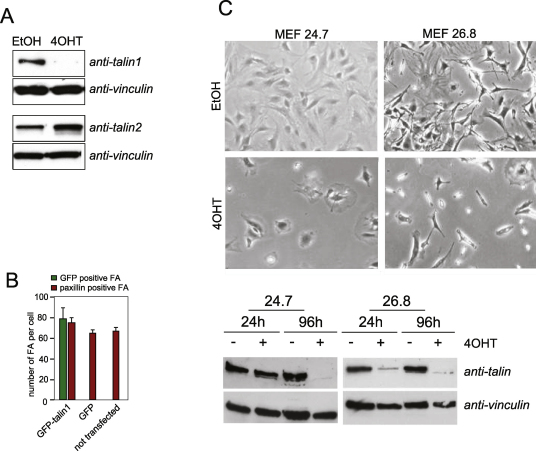
Analysis of MEFs for talin structure function studies. (A) Western blots of *Tln1*^fl/fl^; CreER/+ MEF lysates 72 h after treatment with 100 nM 4-hydroxy tamoxifen (4OHT) or ethanol (EtOH) probed with talin 1 and 2 specific antibodies with vinculin as a loading control. (B) Numbers of GFP- or paxillin-containing FA in *Tln1*^fl/fl^; CreER/+ MEF 72 h after treatment with 100 nM 4OHT and either untransfected or transfected as shown. Results are expressed as mean ± s.e.m. (C) Different batches of primary MEFs differ in their phenotype before and after treatment with 4OHT. (Top panels) Brightfield images of two different *Tln1*^fl/fl^; CreER/+ MEF lines 96 h after treatment with either ethanol (EtOH) or 100 nM 4OHT. (Bottom panels) Western blot of lysates of same MEF lines 24 h or 96 h after treatment with either ethanol (−) or 100 nM 4OHT (+). Vinculin was used as a loading control.

To explore the relationship between talin1 structure and function, we initially sought to establish a talin1 null cell line. Conventional talin1 knockout mice die around 8.0 dpc (Monkley et al., 2000) making it impossible to derive fibroblasts from this source. We therefore generated a mouse line carrying both conditional talin1 and inducible CreER alleles. While treatment of fibroblasts derived from these mice with 4-hydroxy-tamoxifen could be used to generate talin1 null cells, the system proved problematic in that talin2 was rapidly upregulated (Fig. S1A), and the cells were still able to assemble paxillin-containing FAs (Fig. S1B). In addition, primary cultures were difficult to transfect, possessed variable cell morphologies both prior to and following loss of talin1 and differed in the timing of talin1 protein depletion following treatment with 4-hydroxy-tamoxifen (Fig. S1C).

### Human umbilical vein endothelial cells (HUVEC) only express talin1

**Fig. S2:**
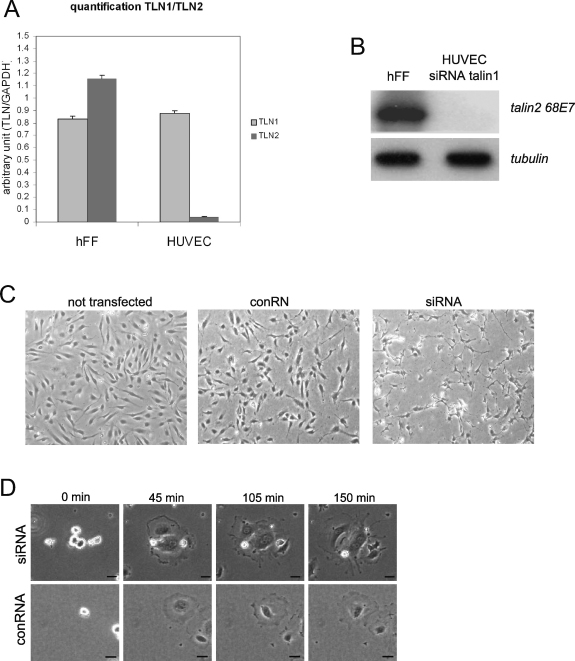
Use of HUVEC for talin1 knockdown. (A) Quantitative RT-PCR of talin1/talin2 expression in either hFF or HUVEC. (B) Western blot of cell lysate from hFFs or HUVECs transfected with talin1 siRNA for 72 h. Tubulin was loading control. (C) Phase micrographs of HUVEC either untransfected or transfected with conRNA or talin1 siRNA for 72 h and replated onto glass for 24 h. (D) HUVEC transfected with either a talin1 siRNA or conRNA were replated on glass coverslips 72 h post transfection. Brightfield images were recorded at the times indicated. Scale bars, 10 μm. For talin 1 siRNA transfected cells, 3 out of the 6 cells shown were able to adhere and spread initially, but they started to arborize after 150 min or could not maintain spreading. Three cells failed to spread at all.

**Fig. 1:**
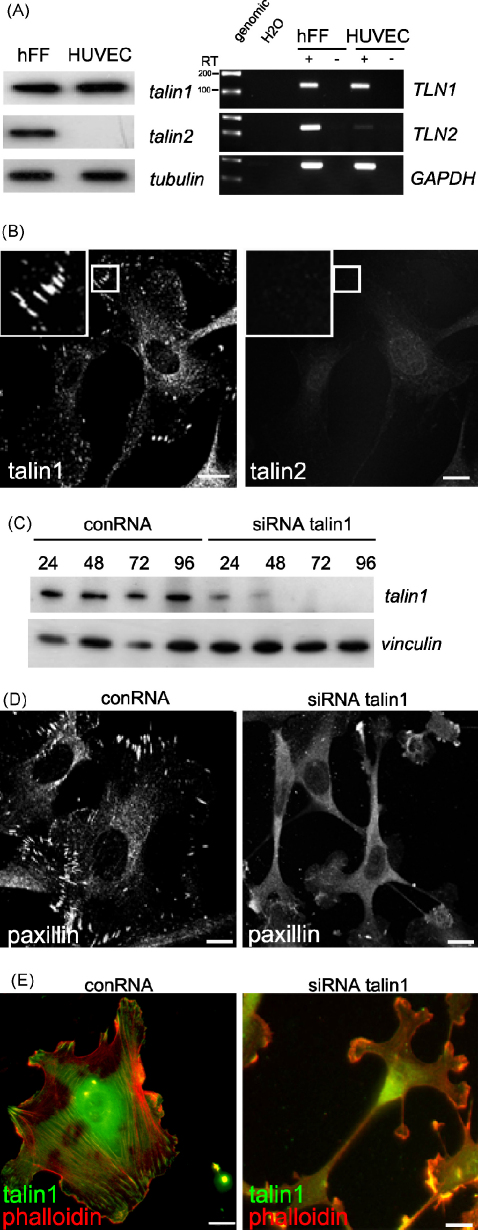
siRNA knockdown of talin1 in HUVEC abolishes FA assembly. (A) Left panel: Western blots of cell lysates from human foreskin fibroblasts (hFF) and HUVEC probed with antibodies specific for talin1 (TA205) and talin2 (mAb121A(53)). Anti-tubulin was used as a loading control. Right panel: RT-PCR amplification of mRNA from hFF or HUVEC (30 cycles) using primers specific for *TLN1* (exons 33–34) and *TLN2* (exons 54–55). H_2_O, control. RT(+) reverse or RT(−) non-reverse transcribed mRNA. (B) Confocal images of HUVEC stained with antibodies specific for talin1 (97H6, left) and talin2 (68E7, right). Scale bar: 10 μm. (C) Western blot of HUVEC treated with a talin1 or control siRNA (conRNA) for 24–96 h; cell lysates were probed with anti-talin1 (97H6), with anti-vinculin as a loading control. (D) HUVEC treated with a talin1 siRNA or conRNA were replated onto glass coverslips 48 h after transfection, and stained 24 h later for paxillin. Scale bar: 10 μm. (E) Epifluorescence image of HUVEC treated as in (D) and stained for F-actin with TexasRed phalloidin (red) and for talin1 with mAb 97H6 (green). Scale bars: 10 μm (For interpretation of the references to colour in this figure legend, the reader is referred to the web version of the article).

Disruption of the talin1 gene in mouse embryos at mid-gestation results in extensive haemorrhage (Monkley et al., unpublished data), and this led us to wonder whether endothelial cells might only express talin1. Analysis of talin1 and talin2 expression in HUVEC using isoform-specific monoclonals showed that as predicted, HUVEC only express talin1 whereas human foreskin fibroblasts express both isoforms (Fig. 1A). Similarly, RT-PCR showed that HUVEC contain little or no talin2 mRNA (Fig. 1A; Fig. S2A), suggesting that the block in talin2 expression is at the transcriptional level. Talin1 was localised to the relatively small FA formed by HUVEC, and no signal was obtained for talin2 (Fig. 1B) even in cells with higher passage numbers (data not shown).

### Knockdown of talin1 in HUVEC leads to a loss of FAs and actin stress fibres

To investigate the role of talin1 in cell spreading and FA assembly, HUVEC were transfected with talin1 siRNAs from Invitrogen, and one was identified that was particularly effective in inducing rapid knockdown of talin1 at the protein level. By 72 h, no talin1 signal could be detected (Fig. 1C) although low levels of talin1 began to emerge after 120 h (data not shown). In contrast, a universal negative control RNA (conRNA) had no effect on talin1 protein levels. Knockdown of talin1 did not lead to a concomitant rise in expression of talin2 (Fig. S2B). HUVEC were therefore transfected with the talin1 siRNA or conRNA and 72 h later were replated onto glass coverslips for a further 24 h after which the cells were fixed and stained for the FA marker paxillin. Subconfluent cells transfected with the conRNA were well spread and contained numerous paxillin-containing FAs, whereas cells transfected with the talin1 siRNA were elongated with few FAs (Fig. 1D and Fig. S2C). There was also a complete loss of actin stress fibres and an accumulation of F-actin in lamellae, whereas in the controls, stress fibres spanned the whole cell and terminated in talin1-positive FA (Fig. 1E).

### Effects of talin1 knockdown on cell spreading and migration

**Fig. 2:**
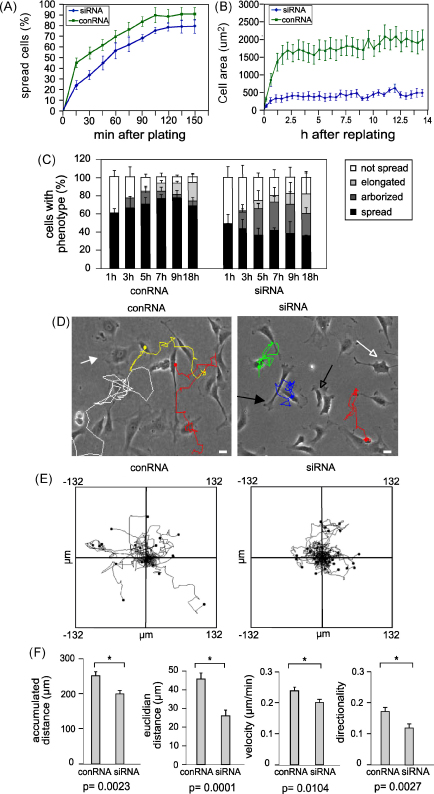
Effects of talin1 knockdown on cell spreading. HUVEC transfected with either the talin1 siRNA or conRNA were replated 72 h after transfection on tissue culture plastic, and imaged every 15 min for the times indicated. (A) The percentage of spread cells was recorded from 3 experiments; mean ± s.e.m. (B) The areas of 12 cells each from two experiments were measured using ImageJ. Results are expressed as mean ± s.e.m. (C) Quantitative analysis of cell morphology at various time points after replating (expressed as mean ± s.e.m.) using the criteria described in Methods. “Spread cell” (white filled arrow in D). “Arborized” (white empty arrow in D). “Elongated” (black filled arrow in D). “Not spread” (black empty arrow in D). *n* = 300 for each time point/sample. (D) HUVEC were imaged every 15 min for 18 h, and three representative cells tracked using the manual tracker tool plugin for ImageJ. Scale bar, 10 μm. (E) Migration plots of HUVEC as in (D). For each plot 25 cells were tracked using the Manual Tracker and Chemotaxis Tool plugins for ImageJ. (F) Quantitation of migration parameters from (E) expressed as mean ± s.e.m. Asterisks represent a significant difference between the two groups. *p*-values are shown under each graph.

To investigate the effect of talin1 knockdown on the rate of cell spreading, HUVEC were transfected with the talin1 siRNA or conRNA, and 72 h later, the cells were replated on tissue culture plastic and imaged over a period of 14 h. Initially, both control and talin1 knockdown cells adhered and began to spread with similar kinetics although the overall proportion of spread cells was slightly reduced in the talin1 knockdown cells (Fig. 2A), and the extent of cell spreading was more variable (Fig. S2D). Quantitative analysis of spread cell area showed that after 15 min there was no significant difference between controls and talin1 knockdown cells (Fig. 2B; conRNA 273.5 μm^2^ ± 36.3; talin1 siRNA 180.6 μm^2^ ± 41.1; *p* = 0.1099). However, after 2 h, controls had reached a near maximal spread cell area of 1645.5 μm^2^ ± 231.3 while the talin1 knockdown cells were markedly smaller (465.0 μm^2^ ± 93.3, *p* = 0.0001), a difference that was maintained for the entire 14 h period (Fig. 2B). To study the effects of talin1 knockdown on cell shape in more detail, the morphology of each cell was assigned to one of four different categories; “spread”, “arborized”, “elongated” and “not spread” (see Materials and methods). Most conRNA-treated cells (∼78%) were well spread 7 h after replating, and only a small number (∼8%) failed to spread. In contrast, although talin1 knockdown cells spread initially (1 h), over time they became elongated or arborized, and by 24 h, only 40% of the cells were spread with 18% remaining unspread (Fig. 2C).

Analysis of cell migration using the Manual Tracking plugin and Chemotaxis tool for ImageJ showed that talin1 knockdown cells showed reduced migration compared to controls (Fig. 2D and E). They travel a significantly shorter trajectory (accumulated distance; *p* = 0.002), and Euclidean distance (the shortest linear distance from the starting point to the end point; *p* = 0.0001) with a slightly reduced velocity (Fig. 2F). As a consequence, the index of directionality defined as the ratio of the Euclidean distance divided by the accumulated distance, is smaller compared to control cells (Fig. 2F). Taken together these data suggest that talin1 is not needed for adhesion of HUVEC or for initial cell spreading, but it is required to maintain the spread phenotype, for persistence of cell movement and for FA assembly.

### Mouse GFP-talin1 rescues the talin1 knockdown phenotype

**Fig. S3:**
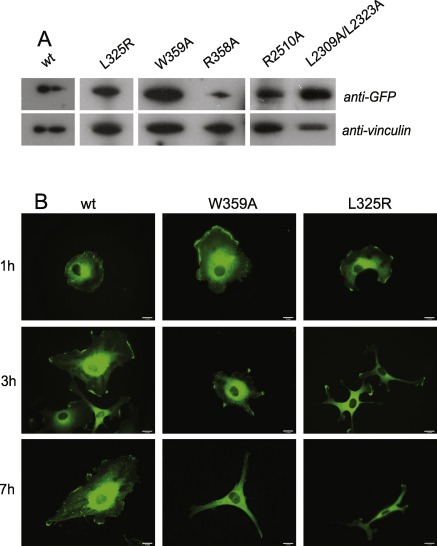
Transfection of HUVEC with talin1 siRNA and talin1-GFP constructs (A) Western blots of lysates of HUVEC transfected with talin1 siRNA and GFP-talin1 constructs as indicated, detected with anti GFP-antibody. Vinculin was used as loading control. (B) Time course of spreading of HUVEC transfected with wild-type GFP-talin1 or constructs with point mutations as indicated, replated 72 h post transfection, fixed after 1 h (top row), 3 h (middle row) or 7 h (bottom row). All cells spread normally during the first hour, after 7 h only the wild-type transfected cells are spread, with the majority of cells expressing mutant GFP-talin1 constructs show an arborized morphology. Scale bars: 10 μm.

**Fig. 3:**
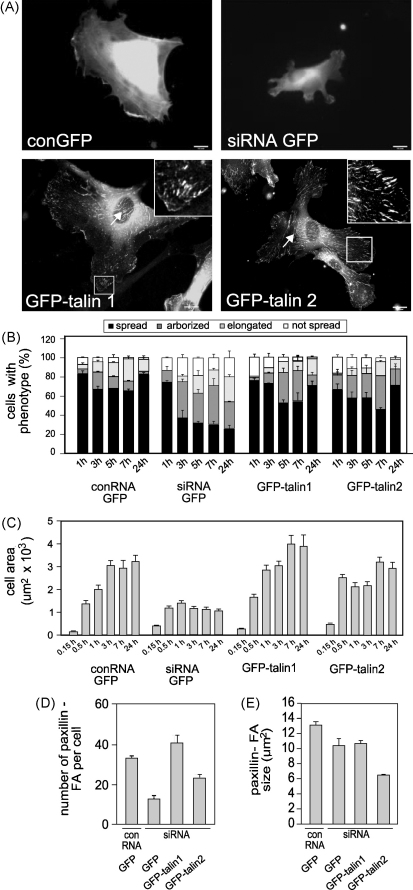
GFP-talin1 or talin2 rescues cell spreading and FA formation in talin1-depleted HUVEC. Cells were transfected with a talin1 siRNA or conRNA plus constructs encoding either GFP alone, mouse talin1-GFP or human talin2-GFP. Cells were replated on glass coverslips 72 h after transfection. (A) Epifluorescence images showing GFP localisation in cells 24 h after replating. (B, C) Time course of changes in cell morphology (B) and cell area (C). (D, E) FA number (D) and size (E) in different cell populations quantified using ImageJ. All results are expressed as mean ± s.e.m. Scale bars: 10 μm.

To confirm that the knockdown phenotype could be rescued by re-expressing talin1, HUVEC were transfected with the human talin1 siRNA together with a plasmid encoding GFP-tagged mouse talin1. After 72 h, cells were replated on glass coverslips, and analysed a further 24 h later. GFP-talin1 expression was confirmed by Western blotting (Fig. S3A). To ensure that phenotypic analysis was conducted on cells expressing similar levels of GFP-talin1, we only imaged those cells that could be visualised at exposure times between 400 and 600 ms on the epifluorescence microscope. Inspection of these images showed that GFP-mouse talin1 fully rescued the spreading defect in talin1 knockdown cells whereas GFP alone was unable to do so (Fig. 3A). Thus, the proportion of GFP-talin1 expressors that were spread (Fig. 3B and C) and the number of paxillin-containing FA (Fig. 3D) was about the same as in cells transfected with the conRNA and GFP. GFP-tagged talin1 localised to peripheral FA as well as to more central fibrillar adhesions (FB) (Fig. 3A arrow), although a cytoplasmic pool of what may be auto-inhibited talin was located around the nucleus. Analysis of the time course of cell spreading showed that talin1 knockdown cells were able to adhere and to spread at least initially (Fig. 3B and C), but this was not maintained, and after 1 h the cells had started to adopt an arborized morphology with multiple protrusions (Fig. 3B) which ultimately resulted in a reduced spread cell area (Fig. 3C). Cells rescued with wild-type GFP-talin1 showed a transient increase in arborized cells (5–7 h), but after 24 h the majority of cells (69%) were well spread. The talin1 knockdown phenotype could also be rescued by GFP-talin2 (Fig. 3A–C) which localised to FA and FB adhesions, although the size and number of paxillin-containing FA was slightly less than in cells expressing GFP-talin1 (Fig. 3D and E).

### Mutations in the F3-integrin-binding domain of talin1 inhibit cell spreading and FA assembly

**Fig. 4:**
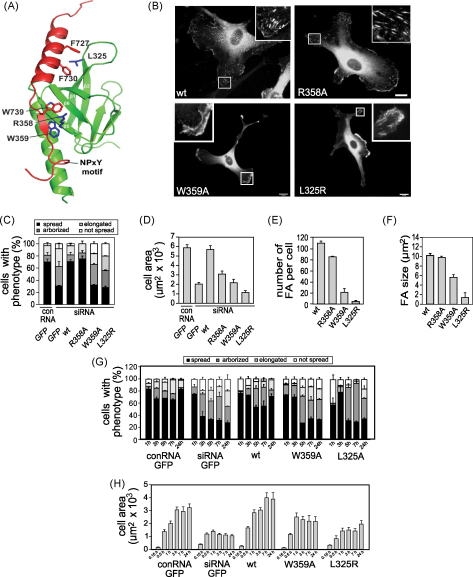
Mutations in the talin1 FERM domain affect cell spreading. HUVEC were transfected with a talin1 siRNA or conRNA plus constructs encoding either GFP alone, wild-type GFP-talin1 (wt) or GFP-talin1 containing point mutations in the FERM domain. Cells were replated on glass coverslips 72 h post transfection and imaged or fixed/stained 24 h later. (A) Structure of the F3 domain of talin1 (green) complexed with the cytoplasmic tail of β3-integrin (red). Side chains of key residues are shown. (B) Epifluorescence images showing GFP localisation 24 h after plating. (C) Cell morphology quantified at 24 h after replating. (D–F) Cell area (D), and the number (E) and size (F) of FA quantified using ImageJ 24 h after replating. (G, H) Time course of cell spreading based on either (G) cell morphology or (H) cell area. All results are expressed as mean ± s.e.m. *p*-values compared to wt at 24 h: Cell area; *p* = 0.0001 (R358A, W359A and L325R). Number of FA; *p* = 0.003 (R358A); *p* = 0..0001 (W359A & L325R). FA size; ns (R358A); *p* = 0.001 (R358A) *p* = 0.006 (L325R). Scale bars: 10 μm (For interpretation of the references to colour in this figure legend, the reader is referred to the web version of the article).

Talin1, and more specifically the talin1 FERM domain, has been shown to play a central role in integrin activation (Nieswandt et al., 2007; Petrich et al., 2007; Tadokoro et al., 2003; Wegener et al., 2007) and clustering (Saltel et al., 2009), and a structural basis for this effect has begun to emerge (Anthis et al., 2009; Garcia-Alvarez et al., 2003; Wegener et al., 2007). We sought to evaluate the conclusions drawn from structural studies by testing the effects of two F3 mutations (R358A and W359A) that dramatically reduce binding of a talin F2F3 polypeptide to the 739-WDTANNPLYDE-749 region of β3-integrin tails (Garcia-Alvarez et al., 2003). As predicted, a full-length GFP-talin1 W359A mutant was unable to rescue the talin1 knockdown phenotype, although surprisingly, cells expressing the GFP-talin1 R358A mutant adopted a near wild-type morphology (Fig. 4B and C). After 24 h, the spread cell area of GFP-talin1 W359A expressors was significantly (*p* = 0.0001) reduced compared to cells expressing wild-type talin1 (Fig. 4D). More detailed time course studies showed that cells expressing the W359A mutant were able to spread during the first 3 h (Fig. 4G and H and Fig. S3B), but they failed to maintain a spread morphology. Moreover, GFP-talin1 W359A failed to support FA assembly, and those FA that did form were smaller and fewer in number than in control cells (Fig. 4B, E and F). In contrast, the R358A mutant localised efficiently to FA (Fig. 4B), and the FA were comparable in number and size to those in controls (Fig. 4E and F). Despite this, the spread cell area of the GFP-talin1 R358A expressors was substantially reduced compared to wild-type (Fig. 4D) indicating that this mutation does have an impact on cell spreading.

**Fig. S4:**
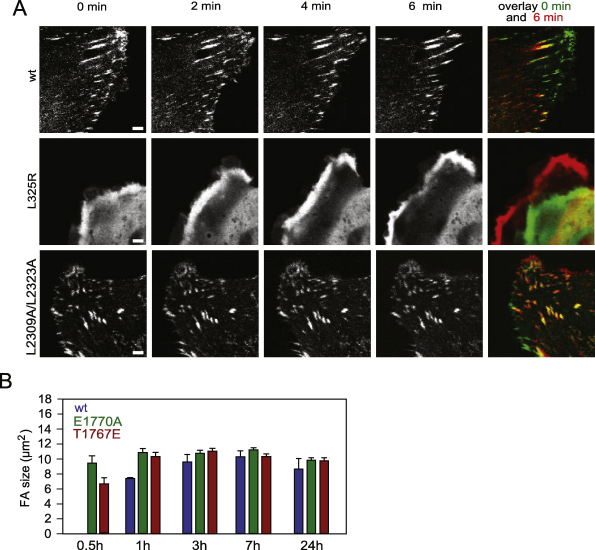
Focal adhesion formation in HUVEC (A) Confocal time-lapse microscopy of HUVEC transfected with talin1 siRNA and the GFP-talin1 constructs indicated 72 h post transfection. The GFP-talin1 L325R mutant localises to highly dynamic lamellae compared to wild-type GFP-talin1. The GFP-talin1 L2309A/L2323A localised to rather stable FAs. The position of the GFP-tagged proteins at 0 min (green) and 6 min (red) are overlaid in the right hand panels. (B) HUVEC were transfected with the talin1 siRNA and either wild-type GFP-talin1 or the GFP-talin1 T1767E or E1770A mutants. FA size was quantified using ImageJ and expressed as mean ± s.e.m. Scale bars: 10 μm.

Leucine 325 is part of a hydrophobic pocket in F3 that interacts with the membrane proximal helical region of β3-integrin tails (Fig. 4A), and a L325R mutation abolishes this interaction and inhibits integrin activation (Wegener et al., 2007). Consistent with the above findings, GFP-talin1 L325R was unable to support cell spreading (Fig. 4B and C), and the cell area was markedly reduced (Fig. 4D). Time course experiments showed that as with the W359A mutation, GFP-talin1 L325R was able to support initial cell spreading (1–3 h), but after that the majority of cells became arborized (Fig. 4G and H and S3B). Similarly, the number and size of FA was dramatically reduced (Fig. 4E and F), and the GFP-talin1 L325R mutant did not localise to FA (Fig. 4B), but instead accumulated in lamellae that were highly dynamic (Fig. S4A). Together, the results show that binding of talin F3 to the NPxY motif and the membrane proximal helix are both required to support cell spreading and FA assembly. The fact that mutations in F3 that compromise integrin binding or activation have such a marked effect on talin1 function raises questions about the significance of the integrin-binding site in the talin rod (Moes et al., 2007; Rodius et al., 2008). Unfortunately, although the structure of the domain containing this site has been determined (Gingras et al., 2009), it has not proved possible to define the binding interface using structural methods, and the role of this site therefore remains unresolved.

### Effect of mutations in the C-ABS of talin1

**Fig. 5:**
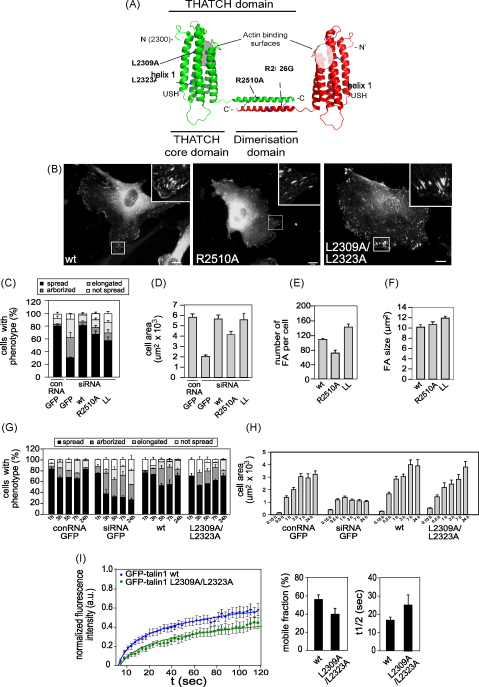
Mutations in the talin1 C-ABS affect cell spreading and FA assembly. HUVEC were transfected with a talin1 siRNA plus constructs encoding either wild-type or GFP-talin1 with point mutations in the C-ABS. Cells were replated on glass coverslips 72 h post transfection, and imaged then fixed/stained 24 h later. (A) Schematic diagram of the dimeric C-ABS of talin1 showing the 5-helix bundle (THATCH domain), the dimerisation domain, and the actin-binding surface. Residues mutated are indicated. (B) Epifluorescence images showing localisation of the various GFP-talin1 constructs 24 h after replating. (C, D) Cell spreading was quantified 24 h after replating onto glass coverslips and was based on cell morphology (C) or cell area (D). (E, F) Quantification of FA size (E) and number (F). (G–H) Time course of cell spreading based on cell morphology (G) or cell area (H). (I) FRAP analysis of FAs in cells expressing either wild-type GFP-talin1 or a GFP-talin1 L2309A/L2323A mutant. Left panel shows averaged curves of normalized fluorescence intensities over time (>10 adhesions from different cells) with regression analysis to illustrate the rate of recovery of GFP-tagged talin1 to the bleached region of FA. Bar charts represent the mobile fraction (middle) and half-life (right) of the GFP-tagged constructs in the FA. All results are expressed as mean ± s.e.m. *p*-values compared to wt at 24 h: Cell area; *p* = 0.004 (R2510A). Number of FA; *p* = 0.0001 (R2510A); *p* = 0.001 (LL). FA size; ns (R2510A); *p* = 0.0004 (LL); FRAP LL; mobile fraction *p* = 0.0001, t1/2 *p* = 0.07. Scale bars: 10 μm.

To investigate the importance of the C-ABS in talin, we analysed the ability of two GFP-talin1 mutants to rescue the talin1 knockdown phenotype. Arginine 2510 is on the exposed highly conserved face of the dimerisation helix (Fig. 5A) and makes salt bridges to neighbouring residues. A C-ABS polypeptide (residues 2294–2541) containing an R2510A mutation is still dimeric, but shows reduced F-actin-binding (∼77% compared to wild-type) (Gingras et al., 2008). Expression of GFP-talin1 R2510A partially rescued the talin1 knockdown phenotype in HUVEC (Fig. 5B) although it was significantly less effective than wild-type GFP-talin1. Thus, there was a small reduction in the percentage of spread cells (Fig. 5C), the spread cell area was significantly reduced (*p* = 0.004, Fig. 5D), and there were fewer FA per cell (Fig. 5E), although FA size was similar to controls (Fig. 5F).

The ability of the C-ABS to bind actin is negatively regulated by helix1 (Smith and McCann, 2007) which is anchored to the rest of the domain through hydrophobic interactions (Fig. 5A), and a L2309A/L2323A double mutant showed a considerable increase (∼154%) in actin-binding compared to wild-type (Gingras et al., 2008). Expression of GFP-talin1 L2309A/L2323A efficiently rescued the talin1 knockdown phenotype as far as cell morphology (Fig. 5B) and the kinetics of cell spreading were concerned (Fig. 5H). However, the cells contained significantly more (Fig. 5E) and slightly larger FA (Fig. 5F), and confocal time-lapse microscopy and FRAP analysis showed that GFP-talin1 L2309A/L2323A positive FA were more stable (Fig. 5I and Fig. S4A), with a lower mobile fraction, and an increased half-life. In summary, the results show that the C-ABS plays an important role in talin1 function despite the presence of several other ABSs in both the talin rod (Hemmings et al., 1996) and the talin head (Lee et al., 2004).

### Mutations that relieve talin auto-inhibition lead to accelerated FA assembly

**Fig. 6:**
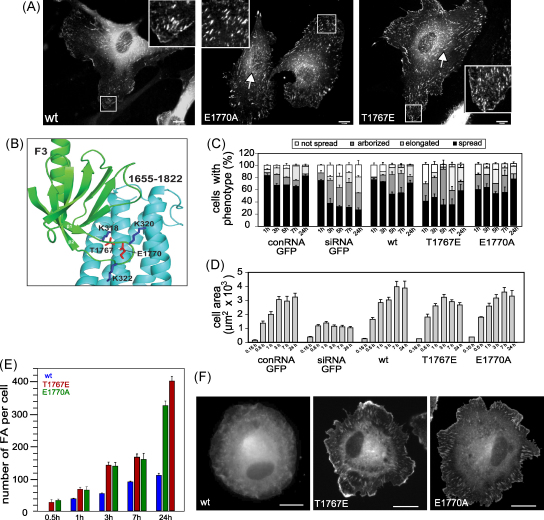
Mutations that relieve talin1 auto-inhibition lead to the rapid assembly of FA. HUVEC were transfected with a talin1 siRNA plus constructs encoding either wild-type GFP-talin1 (wt) or GFP-talin1 rod domain mutants (T1676E or E1770A) that disrupt talin auto-inhibition. Cells were replated on glass coverslips 72 h post transfection, and imaged then fixed/stained 24 h later. (A) Epifluorescence images showing GFP-talin1 localisation 24 h after plating. (B) Diagram of the intramolecular interaction between the F3 FERM domain (green) and residues 1655–1822 in the talin rod (blue). (C, D) Time course of cell spreading following replating onto glass coverslips based on cell morphology (C) or cell area (D). (E) Time course of FA formation. (F) Epifluorescence images of cells 30 min after replating showing localisation of the GFP-talin1 constructs. All results are expressed as mean ± s.e.m. *p*-values compared to wt at 24 h: Cell area; *p* = 0.4 (T1767E, E1770A). Number of FA; *p* = 0.0001 (T1767E, E1770A). Scale bars: 10 μm (For interpretation of the references to colour in this figure legend, the reader is referred to the web version of the article).

Talin can adopt an auto-inhibited conformation due to an interaction between the N-terminal F3 FERM domain and a C-terminal domain in the talin rod (residues 1655–1822) that masks the integrin-binding site in F3 (Goksoy et al., 2008; Goult et al., 2009a) (Fig. 6B). We have identified several mutations in the talin rod that abolish this interaction (Goult et al., 2009a), and we therefore studied the effects of two of these mutations on cell spreading and FA formation. HUVEC transfected with the talin1 siRNA and expressing either GFP-talin1 T1676E or GFP-talin1 E1770A were well spread (Fig. 6A) although the T1676E mutation provoked a transient increase in the number of arborized cells at 5 h (Fig. 6C). However, the most striking effect of these mutations was the increased rate of FA formation, with the first FA appearing as early as 30 min after replating, while in cells transfected with wild-type GFP-talin1, the first FA were only observed after 1 h (Fig. 6E and F). The number of FA continued to increase, and after 24 h there were 3 times more FA in cells expressing either the T1676E or the E1770A mutant than in controls (Fig. 6E and F), although FA size was not affected (Fig. S4B). Both these GFP-talin1 mutants localised to peripheral FA as well as FB-like adhesions (Fig. 6A arrows) whereas wild-type GFP-talin1 was predominantly localised in FA. The results indicate that pathways that regulate the talin head/rod interaction must play a key role in controlling the rate and extent of FA assembly.

## Discussion

Analysis of talin function using constitutive or conditional talin1 knockout cells has been severely limited by the rapid upregulation of talin2, which appears to compensate for loss of talin1. Therefore, the discovery that HUVEC only express talin1 mRNA and protein, and that siRNA depletion of talin1 does not lead to upregulation of talin2 represents a significant breakthrough. Moreover, talin1 knockdown resulted in a robust cellular response i.e. reduced cell spreading and FA assembly, a phenotype that was rescued by the expression of mouse GFP-talin1. The major talin2 promoter is embedded in a CpG island (Debrand et al., 2009), and we presume that the promoter is silenced in endothelial cells by methylation. Indeed, it seems that other cells of the haematopoietic lineage such as platelets (Nieswandt et al., 2007; Petrich et al., 2007), megakaryocytes (Wang et al., 2008), and dendritic cells (Lammermann et al., 2008) also only express talin1 whereas most other cell types express both talin isoforms. However, the role of each of the two isoforms is unclear. While expression of talin2 rescues the talin1 knockdown phenotype in HUVEC, talin1 null mouse embryos die at gastrulation (Monkley et al., 2000) even though RT-PCR-data (Debrand et al., 2009) and Western blotting show that talin2 is expressed in mouse ES cells. In contrast, talin1 and talin2 clearly have overlapping functions in skeletal muscle, and knockout of either gene alone gives rise to only a mild dystrophic phenotype, whereas knockout of both genes leads to a block in myogenesis and is perinatal lethal (Conti et al., 2008, 2009). Interestingly, talin2 co-localises with the muscle-specific β1D integrin splice variant in myotendinous junctions (Conti et al., 2009), and the talin2 F3 FERM domain binds β1D tails with significantly higher affinity than the talin1 F3 domain (Anthis et al., 2009). This may stabilise the junction against the substantial forces exerted on it during muscle contraction, a hypothesis supported by the finding that muscle-specific talin2 knockout mice develop a dystrophic phenotype earlier than talin1 knockout mice (Conti et al., 2008, 2009). In brain, talin2 is the predominant isoform (Debrand et al., 2009), but although it is enriched in nerve growth cones (Pertz et al., 2008), talin2 gene trap mice (Chen and Lo, 2005) and those with a complete disruption of the talin2 gene (Debrand et al., unpublished data) are viable and fertile.

The finding that talin1-depleted HUVEC can initiate cell spreading on plastic or glass surfaces in the presence of serum is consistent with the report that talin-depleted cells can initiate spreading on fibronectin but that this spreading cannot be maintained in the absence of talin (Zhang et al., 2008). The authors showed that talin was not required for the Src family kinase (SFK) signalling events that initiate actin polymerisation and early edge protrusion, but talin was essential for β1-integrin activation, FAK signalling, FA assembly and the exertion of force on the substrate. In the absence of talin, the rate of myosin II-driven rearward flow of actin was markedly elevated and probably contributed to the eventual rounding up of the cells. The cellular response to talin1-depletion in HUVEC was less severe and many of the cells adopted an arborized or elongated morphology, although there was a dramatic reduction in spread cell area as well as the number of FA and actin stress fibres. Cell spreading mediated by β3-integrin has been shown to be dependent on binding of SFK to β3-tails but not on talin binding (Arias-Salgado et al., 2005). Since the major integrin in FA in HUVEC is αVβ3 (Jones et al., 2009), our results suggest that cell spreading in talin1-depleted HUVEC is supported by “outside-in-signalling” via the β3-integrin/SFK pathway.

**Fig. 7:**
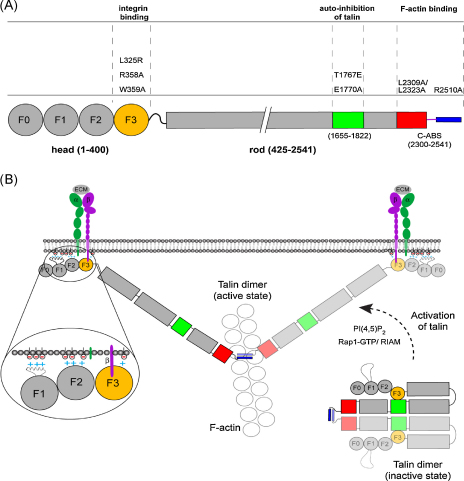
Interactions between talin and its binding partners in FA. (A) Mutations analysed in this study mapped on to the domain structure of talin. (B) Clusters of basic residues in the N-terminal talin1 F1, F2 and F3 FERM domains are shown interacting with acidic membrane phospholipids whilst the F3 domain binds β-integrin tails. The FERM domain is linked to the rod by a flexible linker. The C-ABS is shown bound to a single actin filament. The auto-inhibited form of talin1 is also shown and possible modes of activation are indicated.

We have exploited the fact that mouse GFP-talin1 can be used to rescue the talin1 knockdown phenotype in subconfluent HUVEC to carry out a comprehensive structure function study on talin1 in a cellular context (Fig. 7A). We started by testing the effects of three mutations in the talin F3 FERM domain that have been shown to compromise integrin binding or activation (Garcia-Alvarez et al., 2003; Wegener et al., 2007*)*. Previous analysis of such mutations has largely been restricted to monitoring effects on integrin activation, and not cellular responses such as cell spreading or FA assembly. Surprisingly, expression of the GFP-talin1 R358A mutant largely rescued the talin1 knockdown phenotype in HUVEC, and the same mutant has recently been shown to support Mn^2+^-induced αVβ3-integrin clustering in B16 melanoma cells (Saltel et al., 2009), and integrin clustering in muscle (Tanentzapf and Brown, 2006). The structure of F3 bound to the β3-integrin tail shows that R358 is part of a pocket that accommodates W739 of the β3-tail (Garcia-Alvarez et al., 2003). The guanadinium group of Arg358, which stacks parallel to the aromatic ring of W739 in the β3-tail, points into solution, and it is likely that its loss would not perturb the rest of the pocket or the structure of the F3 domain. Therefore, although the R358A mutation totally ablated binding of a β3-tail peptide to F2F3 in vitro, it is likely that in the context of full-length talin expressed in the cell, other interactions between the talin head, integrin tails and acidic membrane phospholipids (Goult et al., 2010; Anthis et al., 2009; Saltel et al., 2009) partially compensate for the effect of the R358A mutation on integrin binding.

In contrast, the GFP-talin1 W359A or L325R mutants markedly affected the ability of talin to rescue the knockdown phenotype. W359 plays a mainly structural role in F3 maintaining the packing between the β5 strand and the C-terminal helix (Garcia-Alvarez et al., 2003), and the W359A mutation might well affect local folding. In addition, W359 and I396 form a hydrophobic surface that interacts with residues 740-DTA-742 of the β3-tail. Surprisingly, a talin F2F3 W359A mutant retained some ability to bind β3-tails whereas the R358A mutation totally abolished binding (Garcia-Alvarez et al., 2003). Therefore, the marked effect of the W359A mutation on the activity of full-length talin1 suggests that it likely affects the conformation of the talin head such that other interactions required for integrin activation are no longer possible. The discrepancy between the effects of the R358A and W359A mutations on integrin-binding in vitro versus their effects in the cell illustrates the importance of analysing the effects of such mutations in a biological setting.

L325 is the first residue in β-strand 2 in the F3 FERM domain, and with the flexible loop between strands S1 and S2 (Fig. 4A) forms part of a hydrophobic pocket that interacts with F727 and F730 in the membrane proximal helix of β3-integrin tails (Wegener et al., 2007). A L325R mutation abolishes the ability of the talin head to activate integrins, although it does not affect binding of F3 to the membrane proximal NPxY motif in vitro, which provides much of the binding energy for the interaction (Wegener et al., 2007). In line with the above data, the GFP-talin1 L325R mutant was unable to rescue the phenotype of talin1-depleted HUVEC. Indeed, the effects of the mutation on cell area and FA number and size were even more pronounced than seen with the W359A mutant. The result shows that binding of the talin F3 domain to β-integrin tails on its own is insufficient to support integrin-mediated events, and that engagement of F3 with the membrane proximal helical region of β-tails is essential to elicit a full biological response. However, it is also apparent that other regions of the talin head are also required for both β1- and β3-integrin activation (Bouaouina et al., 2008), and we have recently identified clusters of basic residues in both the F1 loop (Goult et al., 2010) and on F2 (Anthis et al., 2009) that bind acidic phospholipids and are required for integrin activation, but not binding. Lysine 322 in F3 has also been shown to play a role in integrin activation (Wegener et al., 2007). Similarly, basic residues on both F2 and F3 have recently been shown to bind PIP_2_ and are required for talin head-mediated Mn^2+^-dependent αvβ3-integrin clustering (Saltel et al., 2009). Wegener and colleagues proposed that the interaction of basic residues on both F2 and F3 with acidic membrane phospholipids contributes to the energy required to stabilise activated integrins (Wegener et al., 2007), and the recent crystal structure of β1D integrin tails complexed to the F2F3 domains of talin2 (Anthis et al., 2009), and the studies of studies of Saltel et al. (2009) are consistent with this idea.

Experiments with talin-depleted cells show that talin is required to couple integrins to the force generating actomyosin contractile machinery (Jiang et al., 2003; Zhang et al., 2008). The best-characterised actin-binding site in talin is at the C-terminus (C-ABS) (Gingras et al., 2008; Smith and McCann, 2007), but its role in integrin-mediated cell spreading and FA assembly has not been investigated in any detail. Indeed, the observation that the centripetal translocation of integrin/talin complexes with F-actin is vinculin dependent led to the suggestion that it is the actin-binding site in the vinculin tail that provides the major link between talin and the actomyosin contractile machinery (Humphries et al., 2007). Here we show that a R2510A mutation in the C-terminal dimerisation helix of C-ABS that reduces its affinity for F-actin by ∼40% (Gingras et al., 2008) compromises the ability of GFP-talin1 to rescue the talin1 knockdown phenotype. Conversely, a L2309A/L2323A mutation that relieves inhibition of C-ABS by helix 1 causes a small but significant increase in the number of FA. Moreover, FRAP experiments show that this mutant exchanges from FAs significantly more slowly than the wild-type GFP-talin1, indicating that it is more tightly associated with FA. How the effect of helix1 on the C-ABS is regulated is not fully understood although recent work suggests that there may be a functional link between the C-ABS and IBS2, the integrin-binding site located in the talin rod (Himmel et al., 2009). It is also possible that force applied to integrin/talin/actin complexes by actomyosin contraction activates the C-ABS by weakening the interaction of helix1 with the 5-helix bundle, as for the VBSs in talin (del Rio et al., 2009) supporting the concept of talin as a mechanotransducer (Roca-Cusachs et al., 2009). In addition, it may be relevant that S2338 in helix1 is phosphorylated in platelet talin (Ratnikov et al., 2005). Whatever the mechanism(s) our results demonstrate that the talin1 C-ABS plays an important role in talin function, although vinculin bound to talin also has the potential to contribute to the interaction between integrin/talin complexes and F-actin.

The results of these and other studies are consistent with a model (Fig. 7B) in which basic residues on the F1, F2 and F3 FERM domains interact with acidic phospholipids while the F3 domain binds to β-integrin tails and activates integrins in the process. The C-ABS plays a key role in coupling talin to the actin cytoskeleton, but since E.M. studies show that it binds along a single actin filament, it cannot account for the ability of talin to cross-link F-actin (Gingras et al., 2008). Perhaps the other ABS in the talin head (Lee et al., 2004) and rod (Hemmings et al., 1996) are important in this regard. The ∼80 residues that link the talin FERM domain to the rod are unstructured and are therefore probably flexible so that the angle between the head and rod is likely to vary. Since talin is about 60 nm long (Molony et al., 1987; Winkler et al., 1997) the distance between the head and the C-ABS could be of the same order. However, residues 1974–2293 contain a second integrin-binding site that also binds acidic phospholipids (Gingras et al., 2009), and we do not exclude the possibility that the whole talin molecule could lie along the membrane.

Talin exists in both an active extended and a more compact auto-inhibited form. Initial indications that this might be the case came from E.M. (Molony et al., 1987; Winkler et al., 1997), and recent NMR studies have shown that the talin F3 FERM domain binds to a 5-helix bundle (residues 1655–1822) in the talin rod masking the integrin-binding site in F3 in the process (Goksoy et al., 2008; Goult et al., 2009a). Although additional intramolecular interactions may contribute to talin auto-inhibition, it is notable that GFP-talin1 constructs carrying mutations (T1767E or E1770A) in the talin rod that abolish F3 binding led to a significant (*p* = 0.0001) increase in the rate of FA assembly and the number of FA compared to controls. The fact that the cells spread more slowly than controls may be due to the overproduction of FA. Interestingly, the talin rod binds to the same loop in F3 that is involved in integrin activation, and mutations in basic residues in the loop that interact with a acidic residues in the talin rod 5-helix bundle (Fig. 7B) enhance the ability of full-length talin to promote Mn2^+^-induced αVβ3-integrin clustering (Saltel et al., 2009). Together, the results suggest that the interaction of F3 with the 5-helix bundle in the talin rod (residues 1655–1822) is a major determinant of the auto-inhibited form of the molecule, and that regulation of this interaction is likely to play an important role in integrin-mediated FA assembly and cell migration (Fig. 7B). Recent studies have shown that the small GTPase Rap1 and its effector RIAM are key regulators of talin activation (Han et al., 2006; Lee et al., 2009; Watanabe et al., 2008), and it will be important to establish how RIAM exerts its effects on talin. In addition, PIP-kinase type1γ (Di Paolo et al., 2002; Ling et al., 2002) and its product PIP2 (Goksoy et al., 2008; Martel et al., 2001) have also been implicated in talin activation. PIP2 inhibits the interaction between F3 and the talin rod (Goksoy et al., 2008), and it has been proposed that PIP2 binds to basic residues in the integrin activation loop disrupting the interaction with the talin rod (Saltel et al., 2009). The relative contributions of these two pathways to talin and integrin activation remain to be investigated.

## Material and methods

### GFP-expression constructs, cell culture and transfection

Mouse talin1 cDNAs and human talin2 cDNA were amplified by PCR and cloned into either pEGFP-N1 (Clontech) or mCherry-N1 (a kind gift from Roger Y. Tsien). Point mutations were introduced by site-directed PCR mutagenesis and authenticated by DNA sequencing. All DNA used for transfection experiments was purified using the Endofree MidiPrep Kit (Qiagen). Human umbilical vein endothelial cells (HUVEC) were purchased from PromoCell at passage 2 and grown according to the manufacturer's instructions in Endothelial Cell Growth Medium 2 (PromoCell) at 37 °C in 10% CO_2_ and cultured on uncoated plastic dishes (VWR International).

### siRNA-mediated knockdown of talin1

For siRNA knockdown of human talin1, three Stealth Select RNAi™ (Invitrogen cat #1299003) were tested, and oligo 804 (seq: CCAAGAACGGAAACCUGCCAGAGUU) was used for all subsequent experiments. A Universal Stealth RNA™ siRNA was used as a negative control (conRNA). Subconfluent HUVEC were trypsinized, washed in PBS, and electroporated (6 × 10^6^ cells/ml) using a Microporator (Invitrogen) according to the manufacturer's instructions, with 100 pmol of talin1 siRNA or controlRNA. Transfection efficiency was ∼98% as determined using fluorescently labelled siRNAs. Cells were grown on tissue culture plastic for 72 h, and replated at a density of 4 × 10^4^ cells on 16 mm glass coverslips (Raymond A. Lamb) for analysis after a further 24 h. Where appropriate, cells were co-transfected with 0.5 μg of plasmid DNA encoding GFP or wild-type/mutant GFP-talin1.

### RNA extraction and RT-PCR

Total cellular RNA was extracted using the RNAeasy Miniprep Kit (Qiagen) according to the manufacturer's instructions. RNA concentrations were quantified at 260/280 nm and treated accordingly with Turbo DNase (Ambion). DNase treated total RNA (1 μg) was reverse transcribed with Superscript III (Invitrogen) and 500 ng of random primers (Promega) in a total volume of 40 μl as recommended by Invitrogen. In addition, non-reversed transcribed controls were generated under the same conditions by replacing the Superscript III enzyme with water. *TLN1* primer pairs from http://primerdepot.nci.nih/gov: left primer sequence (exon 33) 5′-TCTCCCAAAATGCCAAGAAC-3′, right primer sequence (exon 34) 5′-TGGCTATTGGGGTCAGAGAC-3′ were used. For *TLN2* the following primers were designed to detect all *TLN2* transcript isoforms: left primer sequence 5′-CTGAGGCTCTTTTCACAGCA-3′, (exon 54) and right primer sequence 5′-CTCATCTCATCTGCCAAGCA-3′ (exon 55). GAPDH primers were as described in Debrand et al. (2009). Amplification was performed in standard conditions at an annealing temperature of 60 °C using primers at 600 nM and PCR Reddy mix (ThermoScientific) in a volume of 15 μl. Expression of *TLN1* and *TLN2* mRNAs was quantified using real-time PCR in a Roche LightCycler with SybrGreen Mix (Fermentas) and the specific primer sets described above. Primers were used at a final concentration of 300 nM in a 25 μl reaction volume. One microlitre of random-primed cDNA was added to each reaction, and each cDNA sample was amplified in triplicate. The absolute quantity of cDNA in each sample was interpolated on a standard curve obtained from a serial dilution of human foreskin fibroblast (hFF) cDNA. *GAPDH* was used as an internal normalization control.

### Western blotting

Cells were lysed in Laemmli buffer, proteins were resolved by SDS-PAGE and blotted to PVDF membranes The following isoform-specific talin monoclonal antibodies were generated during the course of this study and will be described in more detail elsewhere: anti-talin1 97H6, anti-talin2 68E7 and 121A(53). A previously characterised anti-talin1 monoclonal, TA205 (Bolton et al., 1997) from Santa Cruz was also used. Other antibodies used were: anti-vinculin F9 (Santa Cruz), anti-paxillin (BD Transduction), anti-actin (Sigma) and anti-alpha-tubulin (Abcam). HRP-coupled anti-mouse and anti-rabbit were from GE Healthcare.

### Immunofluorescence and microscopy

Transfected HUVEC were cultured for 24 h on glass coverslips, fixed in 3.5% formaldehyde in PBS-ME (containing 3 mM MgCl_2_ and 3 mM EGTA) for 10 min at room temperature, permeabilized with 0.2% Triton X-100 in PBS-ME for 5 min and stained for F-actin with Alexa 647-phalloidin (1:200) or anti-paxillin (Sigma, 1:100). Talin1 and talin2 were visualised with monoclonal antibodies 97H6 and 68E7, respectively. In this case, and for visualising F-actin with an anti-actin antibody (Sigma, 1:150), cells were fixed and permeabilized in one step with ice-cold methanol for 1 min. Cells were then incubated with 2.5% normal goat serum and 2.5% normal mouse serum for 15 min before staining in 1% BSA in PBS-ME. Alexa-488 or Alexa-594 coupled secondary antibodies (Molecular Probes) were used at a dilution of 1:200. Epifluorescence images were taken with a 40x oil immersion objective on an inverted Nikon TE300 microscope equipped with a Hamamatsu ORCA-ER digital camera and an X-cite 120 fluorescence illumination system controlled by Improvision's Openlab software. For time-lapse experiments, the temperature was kept at 37 °C in an atmosphere containing ∼5% CO_2_. For confocal laser-scanning microscopy, either a Leica TCS SP5 system consisting of a Leica DMI-6000 CS inverted microscope or an Olympus FV1000 system with an inverted IX81 motorized microscope was used with the following emission settings: 500–550 nm for 488 nm excitation (for GFP and FITC), 570–650 nm for 561 nm excitation (TexasRed and mCherry) and 660–755 nm for 633 nm excitation (Alexa647). For time-lapse experiments the cells were plated on 35 mm μ-slides (ibidi GmbH) and phenol-red-free Endothelial Cell Growth Medium 2 (PromoCell) was used.

### FRAP analysis

Experiments were performed using a Leica TCS SP5 system attached to a Leica DMI-6000 CS inverted microscope equipped with a 63× oil immersion objective (NA 1.4). Stage and objective were placed in an environment chamber pre-equilibrated to 37 °C. The cells were kept at 5% CO_2_ on the stage. The image acquisition was performed using the Leica FRAP Wizard using bidirectional scanning at 400 Hz in an image format of 1024 × 1024 pixel. The bleaching set up consisted of a pre-bleach period of 5 frames at 5% laser power at 488 nm, then an area of 2 μm × 2 μm was bleached with a laser intensity of 70% for 5 frames, followed by a post-bleach period for 40 frames at 5% laser intensity. The emission settings were 500–550 nm and a 6× zoom was used. Time interval between the start of each image during pre- and post-bleaching was 3 s. Background values were determined and subtracted from all raw data before further analysis with MS Excel. The mobile fraction was calculated as the maximum intensity of recovery after curve fitting, and the t1/2 as the period of time elapsed until the curve reached half its maximum.

### Image and data analysis

Images were analysed using ImageJ (NIH) by converting them to 8-bit images. Following thresholding, cell area, FA/FB number and size were measured by setting the size limits of the measured particles to 20–500 pixels (0.3–70 μm^2^). For each experiment 30 cells were analysed, and the experiment was conducted in triplicate. Cell morphology was assessed on 100 cells/experiment and experiments were conducted in triplicate. The following criteria were used: “spread”: area of cytoplasm is three times bigger than the area of the nucleus; “arborized”: cell with more than 5 prominent protrusions or more than 3 axes, “elongated”: cell width at least 5 times bigger than cell length, “not spread”: area of cytoplasm is as big or smaller than three times the area of the nucleus. A two-tailed unpaired Student's *t*-test was performed to test for significance in all experiments.
